# Antibacterial activity of chitosan against *Burkholderia pseudomallei*

**DOI:** 10.1002/mbo3.534

**Published:** 2017-11-27

**Authors:** Watcharaporn Kamjumphol, Pisit Chareonsudjai, Sorujsiri Chareonsudjai

**Affiliations:** ^1^ Department of Microbiology Faculty of Medicine Khon Kaen University Khon Kaen Thailand; ^2^ Melioidosis Research Center Khon Kaen University Khon Kaen Thailand; ^3^ Department of Environmental Science Faculty of Science Khon Kaen University Khon Kaen Thailand; ^4^ Biofilm Research Group Khon Kaen University Khon Kaen Thailand

**Keywords:** antibacterial activity, *Burkholderia pseudomallei*, chitosan, damage bacterial cell membrane, transmission electron microscope

## Abstract

The ability of *Burkholderia pseudomallei* to persist and survive in the environment is a health problem worldwide. Therefore, the antibacterial activities of chitosan against four environmental isolates of *B. pseudomallei* from soil in Khon Kaen, Thailand, were investigated. Antibacterial activities were assessed by a plate count technique after treatment with 0.2, 0.5, 1, 2 or 5 mg ml^−1^ chitosan for 0, 24 and 48 hr. Chitosan at 5 mg ml^−1^ completely killed all four *B. pseudomallei* isolates within 24 hr, whilst 2 mg ml^−1^ chitosan lowered the viability of *B. pseudomallei* by 20% within the same time span. Chitosan may act by disruption of the cell membrane, releasing intracellular components that can be detected spectrophotometrically at 260 and 280 nm. Transmission electron microscopy inspection of chitosan‐treated *B. pseudomallei* revealed damage to the bacterial membranes. This study demonstrated the effective antibacterial activity by chitosan against *B. pseudomallei*. Chitosan causes disruption of the bacterial cell membrane, release of intracellular constituents and cell death. This study revealed the inhibitory potential of chitosan for mitigating *B. pseudomallei* occurrences.

## 1 INTRODUCTION

The environmental gram‐negative bacterium *Burkholderia pseudomallei* has been recognized as a causative agent of melioidosis since 1911 (Whitmore & Krishnaswami, 1912). This infectious disease remains a health concern worldwide, particularly in Southeast Asia and northern Australia, where high environmental prevalence of the bacterium is correlated with a high incidence of melioidosis cases (White, 2003; Cheng & Currie, 2005; Limmathurotsakul & Peacock, 2011; Wiersinga, Currie, & Peacock, 2012). Melioidosis can be naturally acquired by inoculation, inhalation or ingestion (White, 2003; Limmathurotsakul & Peacock, 2011; Wiersinga et al., 2012). The disease has diverse clinical manifestations, leading to diagnostic delays and difficulties, and is intrinsically resistant to a wide range of antimicrobials. Relapsing melioidosis is common, causing high mortality (Wiersinga et al., 2012; Limmathurotsakul et al., 2014, 2016).

*Burkholderia pseudomallei* is a tough organism with extraordinary persistence either in environmental or laboratory settings (Tong, Yang, Lu, & He, 1996; Chen, Chen, Kao, & Chen, 2003; Inglis & Sagripanti, 2006; Pumpuang et al., 2011). Some environmental conditions are known to be inimical to *B. pseudomallei*. For example, 10% and 40% calcium oxide effectively inhibits its growth (Na‐ngam, Angkititakul, Noimay, & Thamlikitkul, 2004). In addition, colony numbers of *B. pseudomallei* were significantly reduced in soil microcosms of pH > 8, soil salinity > 1% NaCl, and C/N ratio > 40:1 (Wang‐Ngarm, Chareonsudjai, & Chareonsudjai, 2014). Our co‐cultivation experiments recently demonstrated that free‐living amoebae isolated from soils in melioidosis‐endemic areas may prey upon *B. pseudomallei* (Noinarin, Chareonsudjai, Wangsomnuk, Wongratanacheewin, & Chareonsudjai, 2016). Also, Boottanun, Potisap, Hurdle, & Sermswan, (2017) recently demonstrated that secondary metabolites from *Bacillus amyloliquefaciens* isolated from soil can lower the numbers of *B. pseudomallei* by 5 log_10_ within 72 hr. Consequently, additional newer and safer antimicrobial compounds have received considerable attention in efforts to mitigate or control the numbers of *B. pseudomallei* in recent years.

Chitosan is a natural biopolymer derived from chitin by deacetylation. It has broad‐spectrum antimicrobial activity against many antibiotic‐resistant microorganisms (gram‐negative and ‐positive) by damaging the bacterial cell membrane (Muzzarelli et al., 1990; Liu, Du, Wang, & Sun, 2004; Raafat & Sahl, 2009; Li et al., 2010; Tao, Qian, & Xie, 2011) without increasing resistance (Ma et al., 2016). Due to its excellent properties of biodegradability and low toxicity to mammalian cells, chitosan has been used to control some microbial plant pathogens for crop protection (Campaniello, Bevilacqua, Sinigaglia, & Corbo, 2008; Lou et al., 2011; Badawy, Rabea, & Taktak, 2014; Jovanovic, Klaus, & Niksic, 2016) and for treatment of infectious agents including *Helicobacter pylori* (Choi, Lee, & Chae, 2014), oral pathogens (Costa, Silva, Pina, Tavaria, & Pintado, 2012; Franca et al., 2014), *Staphylococcus aureus* (Han et al., 2016), *Escherichia coli* (Liu et al., 2006; Li et al., 2010; Jeon, Oh, Yeo, Galvao, & Jeong, 2014; Gyliene et al., 2015). Antimicrobial activity of chitosan against highly pathogenic bacteria (*Aeromonas hydrophila, Edwardsiella ictaluri,* and *Flavobacterium columnare*) of warm‐water cultured finfish has also been demonstrated (Yildirim‐Aksoy & Beck, 2017). Chitosan acts by interaction of its positively charged glucosamine groups with the negatively charged bacterial or fungal cell membranes, leading to leakage of intracellular components (Raafat & Sahl, 2009; Kong, Chen, Xing, & Park, 2010).

We know of three reports on antibacterial activity and mechanism of action of chitosan solutions against members of the genus *Burkholderia*. One study concerned *B. seminalis*, the apricot fruit‐rot pathogen, a member of the *B. cepacia* complex (Lou et al., 2011). Another study on members of the same complex was conducted in sputum from cystic fibrosis (CF) patients in China (Fang et al., 2010). The third report focused on the multidrug‐resistant *B. cenocepacia* (Ibrahim et al., 2014). Chitosan can lethally damage bacterial cell membranes leading to the leakage of proteins, nucleic acids and other intracellular components. To date, there has been no research on the antibacterial activity of chitosan against *B. pseudomallei*. This, therefore, was the aim of our study. Assays of the integrity of the bacterial cell membrane were done and transmission electron microscopy observations used to elucidate the mechanism of the antibacterial activity of chitosan against *B. pseudomallei*.

## 2 MATERIALS AND METHODS

### 2.1 Materials

Chitosan with degrees of *N*‐deacetylation not less than 85%, practical grade, from crab shells, was purchased from Sigma‐Aldrich (St. Louis, MO, USA). Chitosan was dissolved in 1% (v/v) acetic acid to produce stock solutions of 10 mg ml^−1^. The pH was adjusted to 5.6 using NaOH and continuous stirring at 160 rpm for 24 hr at room temperature. This was followed by autoclaving at 121°C for 20 min. The stock chitosan solution was diluted to the desired concentrations in sterile deionized water of pH 5.6. In the control treatment, sterile deionized water of pH 5.6 was substituted for chitosan stock.

### 2.2 Bacterial strains

Four strains of environmental *B. pseudomallei* (ST‐39, MBPE228, MBPE230, and MBPE232) isolated from soil in Khon Kaen, Thailand (Suebrasri, Wang‐ngarm, Chareonsudjai, Sermswan, & Chareonsudjai, 2013), were used in this study. *Escherichia coli* and *Staphylococcus aureus* were also used in parallel for comparison. The bacteria from −80°C glycerol stocks were cultured on Luria‐Bertani (LB) agar at 37°C for 24 hr. A single colony of each bacterial strain was inoculated into LB broth and incubated at 37°C for 18 hr with shaking at 200 rpm. The bacteria were harvested by centrifugation at 2,810*g* for 15 min at 4°C and washed twice with sterile distilled water. Thereafter, the bacterial cells were resuspended in sterile distilled water (pH 5.6) and density adjusted to achieve OD_600_ of 0.6 (approximately 10^8^ colony forming unit (cfu) ml^−1^) for the antibacterial activity assay.

### 2.3 Antibacterial activity of chitosan against *B. pseudomallei*

Final concentrations of chitosan used in our experiments were 0.2, 0.5, 1, 2, and 5 mg ml^−1^. Bacterial suspension was adjusted to approximately 10^8 ^cfu ml^−1^. One hundred microliter of bacterial suspension was added to chitosan solutions in a 96‐well plate and incubated for 0, 24 or 48 hr at 37°C with agitation at 180 rpm as previously described by Lou and others (Lou et al., 2011). The chitosan‐treated bacteria were thereafter serially diluted and 10 μl of each dilution was plated on LB agar in 10 replicate (Herigstad, Hamilton, & Heersink, 2001). After incubation at 37°C for 24 hr, the viable bacteria were enumerated based on numbers of colony‐forming units. The percentage of killing was calculated using the formula [1‐(log10 sample/log10 inoculum)]× 100 (Fang et al., 2010). Each experiment was carried out in duplicate in three independent experiments.

### 2.4 Integrity of cell membranes

Cell‐membrane integrity of the treated *B. pseudomallei* was examined by determination of the absorption values of released material at 260 nm (A_260_) and 280 nm (A_280_) (Wang et al., 2012). The bacterial cells were harvested, washed twice and resuspended in sterile distilled water of pH 5.6 and adjusted to an OD_600_ of 0.6. The chitosan solutions were added to the bacterial suspension to give final chitosan concentrations of 0.5, 1, 2, and 5 mg ml^−1^. The release over time of materials absorbing at 260 and 280 nm was recorded with a lambda 35 uv/vis spectrophotometer (Ultraspec^®^ pro, Amersham, Biosciences). Triton® X‐100 (Merck, KGaA, Darmstadt, Germany) at a concentration of 0.01% (v/v) was used as a positive control. Each experiment was carried out in duplicate in three independent experiments.

### 2.5 Transmission electron microscopy

One milliliter of *B. pseudomallei* ST‐39 suspension of approximately 10^8 ^cfu ml^−1^ was added into sublethal chitosan solutions with final concentrations of 1 and 2 mg ml^−1^. After incubation at 37°C, 180 rpm for 24 hr, the suspension was centrifuged, washed twice with sterile distilled water and fixed with 2.5% (v/v) glutaraldehyde (EM grade; EMS, Electron microscopy Sciences, USA) in 0.1 mol L^‐1^ phosphate buffer (PBS, pH 7.4) at 4°C for 2 hr. Subsequently, the samples were washed three times with 0.1 mol L^‐1^ PBS followed by postfixing with 1% (w/v) OsO_4_ in 0.1 mol L^‐1^ PBS for 2 hr at room temperature. After three washes with the same buffer, the samples were dehydrated by a graded series of ethanol solutions (70%, 80%, 90% and 100%) as previously described by Lou and others (Lou et al., 2011). The samples were then embedded in Spurr's resin and sectioned with an ultramicrotome (Leica EM UC7 ultramicrotome, Germany) at room temperature. Thereafter, the sections were double‐stained with saturated uranyl acetate and lead citrate. The grids were examined with a transmission electron microscope (Hitachi HT7700, Japan) at an operating voltage of 80 kV.

## 3 RESULTS

### 3.1 Antibacterial activity of chitosan against *B. pseudomallei*

**Figure 1:**
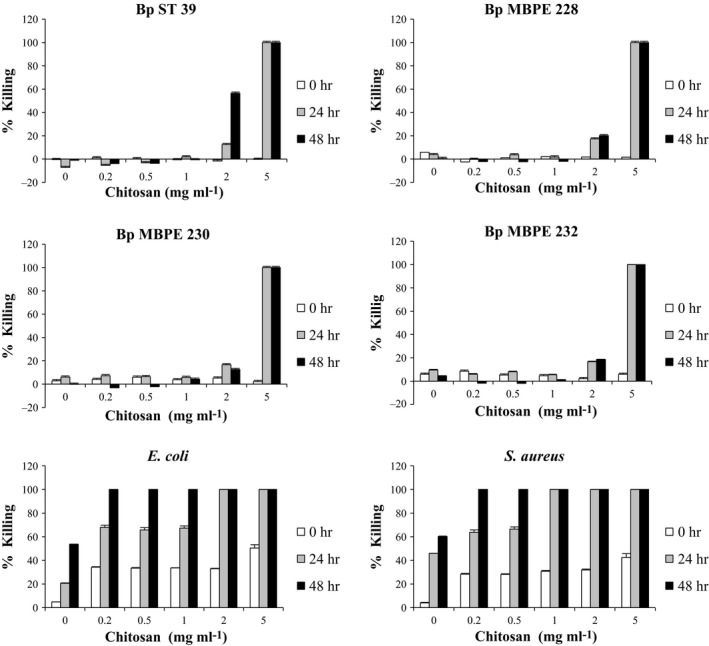
Antibacterial activity of chitosan (0–5 mg ml^−1^) against *B. pseudomallei* ST 39, MBPE 228, MBPE 230, MBPE 232, *E. coli* and *S. aureus* after 0, 24, and 48 hr. The killing percentage was determined by comparing the numbers of viable bacterial cells between experimental and control groups. Data are presented as the mean and standard deviation of three independent experiments performed in duplicate

The antibacterial activity of chitosan against *B. pseudomallei* strains ST 39, MBPE 228, MBPE 230 and MBPE 232 is shown in Figure 1. Chitosan at 5 mg ml^−1^ exhibited complete killing of all 4 strains of *B. pseudomallei* within 24 hr, whereas chitosan at 2 mg ml^−1^ produced only about 20% bactericidal activity after 24 and 48 hr. At concentrations lower than 2 mg ml^−1^, there was little effect on bacterial viability. Antibacterial activity of chitosan against *B. pseudomallei* was therefore concentration‐dependent. The most effective inhibition activity of chitosan against *B. pseudomallei* was at 5 mg ml^−1^. Remarkably, the antibacterial activity of chitosan against *B. pseudomallei* was much less than against *E. coli and S. aureus* (Figure 1).

### 3.2 Integrity of *B. pseudomallei* cell membranes

**Figure 2:**
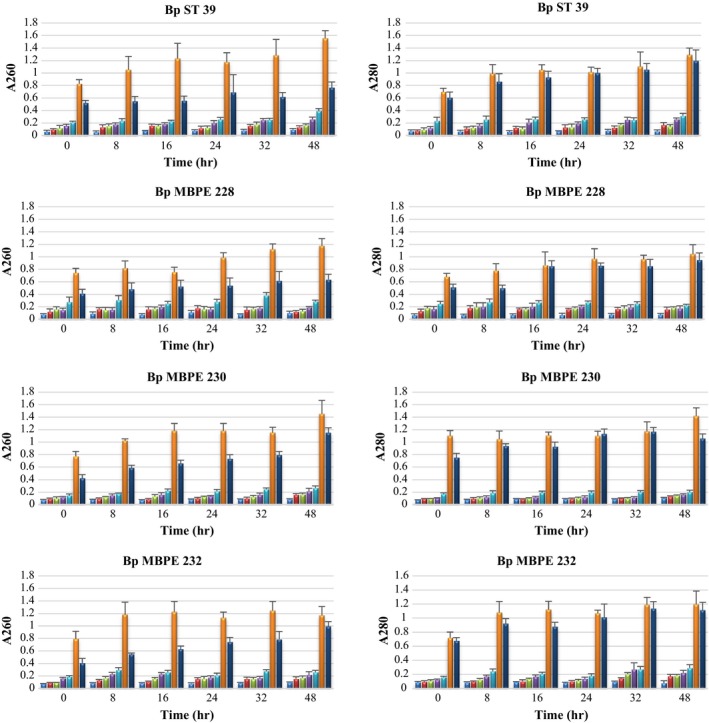
Release of cell materials absorbing at 260 nm (left panel) and 280 nm (right panel) from *B. pseudomallei* ST 39, MBPE 228, MBPE 230 and MBPE 232 treated with chitosan at 0 (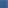), 0.2 (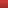), 0.5 (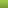), 1 (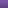), 2 (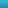), 5 (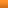) mg ml^−1^ and 0.01% Triton‐X 100 (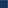). Data are presented as the mean and standard deviation of three independent experiments performed in duplicate

To illustrate the antimicrobial mechanism of chitosan against *B. pseudomallei*, the integrity of cell membranes in the treated *B. pseudomallei* was assessed using the release of intracellular materials as an indicator. The A260 and A280 values, estimating nucleic acid and protein released from treated *B. pseudomallei* treated with 0–5 mg ml^−1^ chitosan for 24 hr, were determined. The positive control used 0.01% Triton X‐100 to lyse all cells. Release of intracellular components was concentration‐dependent up to the highest concentration used in this study (5 mg ml^−1^) (Figure 2). Notably, the damage to bacterial cell membranes by chitosan is in agreement with the results of the bactericidal activity investigation.

### 3.3 Transmission electron microscopy

**Figure 3:**
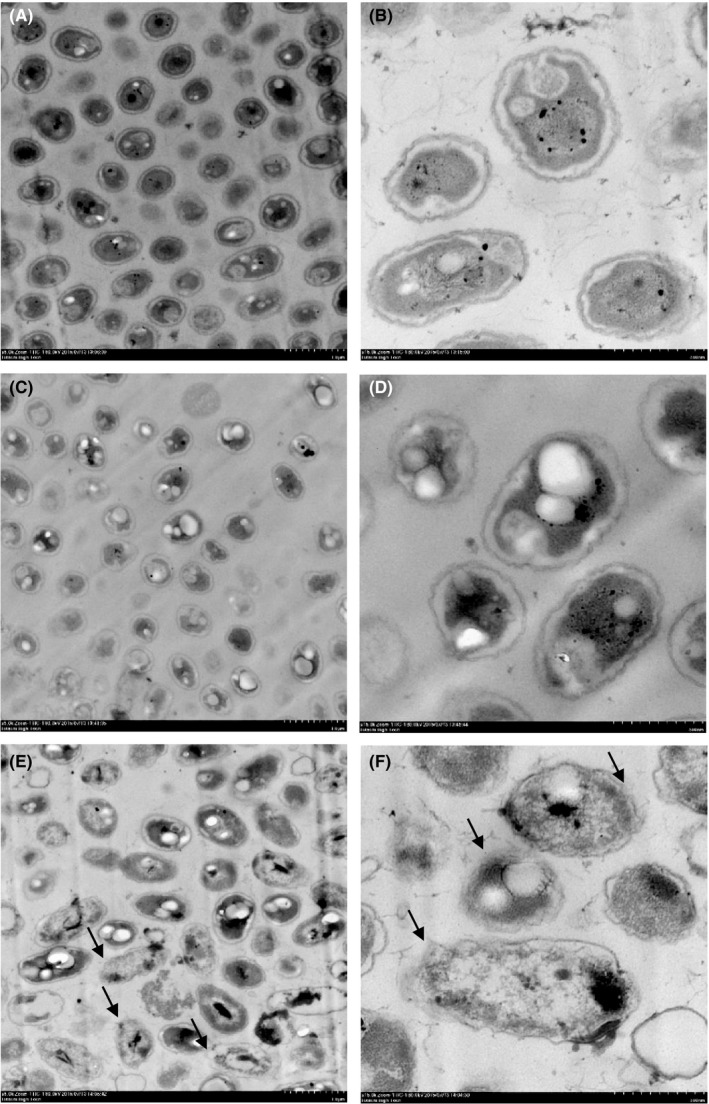
Transmission electron microphotographs of environmental *B. pseudomallei* strain ST 39: untreated bacteria (A and B), bacteria treated with chitosan at concentrations of 1 mg ml^−1^ (C and D), and 2 mg ml^−1^ (E and F) for 24 hr, amplified 5,000 (left panel) and 15,000 (right panel). Arrows indicate cell membrane damage

The effects of chitosan on the morphology of *B. pseudomallei* ST 39 cells, untreated or treated with sublethal chitosan concentrations of 1 or 2 mg ml^−1^ for 24 hr, were examined by transmission electron microscopy at magnifications of 5,000 (left panel) and 15,000 (right panel) (Figure 3). Untreated bacterial cells exhibited a smooth surface without any notable ruptures (Figures 3A and 3B). Cells treated with 1 mg ml^−1^ chitosan were similar to this in morphology (Figures 3C and D). However, the membranes of bacterial cells treated with 2 mg ml^−1^ chitosan exhibited some disruption, as shown in Figures 3E and F. Some cells had become irregular in shape and parts of the cell wall were shattered, possibly leading to leakage of cellular cytoplasmic contents.

## 4 DISCUSSION

*Burkholderia pseudomallei* is a soil‐dwelling saprophyte that can be acquired from the environment, leading to a fatal infection termed melioidosis (Cheng & Currie, 2005; Limmathurotsakul & Peacock, 2011; Wiersinga et al., 2012). The remarkable ability of *B. pseudomallei* to survive for months to years in the environment may increase the risk of transmission to humans (Inglis & Sagripanti, 2006; Kamjumphol, Chareonsudjai, Taweechaisupapong, & Chareonsudjai, 2015). In light of this, attempts have been made to lower the bacterial population to decrease the risk of their transmission to humans (Na‐ngam et al., 2004; Wang‐Ngarm et al., 2014; Boottanun et al., 2017).

In this study, we demonstrated that the greatest killing activity of all four environmental *B. pseudomallei* strains occurred when bacteria were exposed to 5 mg ml^−1^ of chitosan. Chitosan at a concentration of 2 mg ml^−1^ inhibited bacterial growth by about 20% within 24 hr. The inhibitory activity of chitosan against *B. pseudomallei* was evidently a function of the concentration of chitosan since the control treatment (sterile deionized water of pH 5.6) had almost no effect on the survival of *B. pseudomallei*. This result is consistent with the optimum pH of 5–8 determined for the survival of *B. pseudomallei* by Tong et al. (1996). Our results are in line with those of Fang and colleagues who demonstrated that chitosan was a potential bactericidal agent against cells of the *B. cepacia* complex isolated from cystic fibrosis patients. In those cases, effective concentrations of chitosan were 10–100 μg ml^−1^ (Fang et al., 2010). Activity of chitosan against *B. cenocepacia*, a multidrug‐resistant pathogen that is difficult to eradicate, has also been demonstrated (Ibrahim et al., 2014). Leaving aside human pathogens, the apricot fruit‐rot pathogen, *B. seminalis*, could be eradicated by treatment with chitosan at 2 mg ml^−1^ (Lou et al., 2011). However, our findings demonstrated that *B. pseudomallei* can withstand higher chitosan concentrations than do other *Burkholderia* species. Chitosan is known to act against bacteria by damaging the bacterial cell integrity and causing cell membrane permeabilization. The positive charge of chitosan is assumed to interact with anionic components such as lipopolysaccharides, phospholipids and bacterial cell‐surface proteins (Chung et al., 2004; Kong et al., 2010). Given that *B. pseudomallei* bears highly species‐specific antigens that can be exploited for diagnosis (Pitt, Aucken, & Dance, 1992; Perry, MacLean, Schollaardt, Bryan, & Ho, 1995; Ho et al., 1997; Dharakul, Songsivilai, Smithikarn, Thepthai, & Leelaporn, 1999), its cell surface structure is presumably different from that of other *Burkholderia* species. Nevertheless, like other members of the genus, *B. pseudomallei* is susceptible to chitosan.

We showed here that chitosan disrupts *B. pseudomallei* cell membranes with the release of intracellular contents resulting in a drastic increase in absorbance at A260 and A280 compared to untreated controls. Damage to the cell membrane was directly observed by transmission electron microscopy in chitosan‐treated *B. pseudomallei*. We demonstrated disruption of the bacterial cell membrane and confirmed the antibacterial activity of chitosan against *B. pseudomallei*. To extend the applicability of our findings, further studies are required to demonstrate that chitosan can control *B. pseudomallei* in soil or, combined with other antimicrobial agents, that chitosan may improve outcomes for melioidosis patients.

In summary, our work has added more information concerning the antibacterial activity of a natural and nontoxic biopolymer, chitosan. At a concentration of 5 mg ml^−1^, chitosan can kill *B. pseudomallei*, the causative agent of melioidosis. The mechanism by which chitosan exerts this effect is damage to the bacterial cell membrane leading to leakage of intracellular components. Chitosan has the potential to limit the numbers of *B. pseudomallei* cells.

## CONFLICT OF INTEREST

The authors have no conflict of interest to declare.
